# The 12-Month Course of ICD-11 Adjustment Disorder in the Context of Involuntary Job Loss

**DOI:** 10.32872/cpe.v2i3.3027

**Published:** 2020-09-30

**Authors:** Louisa Lorenz, Andreas Maercker, Rahel Bachem

**Affiliations:** a Department of Psychology, University of Zurich, Zurich, Switzerland; Philipps-University of Marburg, Marburg, Germany

**Keywords:** adjustment disorder, ICD-11, job loss, prevalence, disorders specifically associated with stress

## Abstract

**Background:**

After its redefinition in ICD-11, adjustment disorder (AjD) comprises two core symptom clusters of preoccupations and failure to adapt to the stressor. Only a few studies investigate the course of AjD over time and the definition of six months until the remission of the disorder is based on little to no empirical evidence. The aim of the present study was to investigate the course of AjD symptoms and symptom clusters over time and to longitudinally evaluate predictors of AjD symptom severity.

**Method:**

A selective sample of the Zurich Adjustment Disorder Study, N = 105 individuals who experienced involuntary job loss and reported either high or low symptom severity at first assessment (t1), were assessed M = 3.4 (SD = 2.1) months after the last day at work, and followed up six (t2) and twelve months (t3) later. They completed a fully structured diagnostic interview for AjD and self-report questionnaires.

**Results:**

The prevalence of AjD was 21.9% at t1, 6.7% at t2, and dropped to 2.9% at t3. All individual symptoms and symptom clusters showed declines in prevalence rates across the three assessments. A hierarchical regression analysis of symptoms at t3 revealed that more symptoms at the first assessment (β = 0.32, p = .002) and the number of new life events between the first assessment and t3 (β = 0.29, p = .004) significantly predicted the number of AjD symptoms at t3.

**Conclusion:**

Although prevalence rates of AjD declined over time, a significant proportion of individuals still experienced AjD symptoms after six months. Future research should focus on the specific mechanisms underlying the course of AjD.

## Abstract (highlights)

- Symptoms of ICD-11 adjustment disorder were highly prevalent among individuals who experienced involuntary job loss up to nine months previously.
- In 30% of the adjustment disorder cases the symptoms persisted beyond the 6-month remission threshold defined in the diagnostic manuals.
- Subsequent life events might complicate recovery from adjustment disorder.
- Mechanisms underlying symptom improvement or exacerbation need to be further studied.

The new description of adjustment disorder (AjD) in the International Classification of Diseases, 11^th^ version (ICD-11) includes the presence of (a) one or a series of psychosocial stressor(s); of (b) preoccupation with the stressor(s); of (c) failure to adapt to the stressor(s); and of (d) significant impairment in personal, family, social, educational, occupational or other important areas in functioning (World Health Organisation [WHO], 2018). In contrast, the Diagnostic and Statistical Manual of Mental Disorders, 5^th^ version (DSM-5) does not define specific symptoms and the diagnosis of AjD is not applicable in the presence of any other mental disorder (American Psychiatric Association [APA], 2013). The usage of AjD based primarily on an exclusion criterion in DSM-5 and earlier ICD-versions has resulted in its usage as a diagnostic rest category with subsyndromal character (Bachem & Casey, 2018; Baumeister & Kufner, 2009). Another difference between the current manuals is that the DSM-5 distinguishes subtypes of AjD, such as depressed mood, anxiety, disturbance of conduct and mixed subtypes (APA, 2013), whereas the ICD-11 does not.

The diagnostic manuals state that the symptoms usually emerge within one (ICD-11) and three (DSM-5) months after the onset of the stressor and that they typically resolve within 6 months, unless the stressor persists for a longer duration (WHO, 2018). This makes AjD a disorder with an essential benign outcome and spontaneous remission by definition. A few studies that investigated readmission rates for AjD cases in clinical settings support this concept (Jäger, Burger, Becker, & Frasch, 2012; Jones, Yates, & Zhou, 2002). However, AjD is also associated with an elevated risk for concurrent or subsequent mental disorders and for suicidality (Casey & Doherty, 2012; Gradus et al., 2010; O’Donnell et al., 2016) and the definition of the 6-months’ period is still based on little to no empirical evidence. In injury survivors, 16% of the participants still met the diagnostic criteria of DSM-5 AjD after twelve months post-injury (O’Donnell et al., 2016). In a representative sample from Germany, a significant proportion of individuals who reported AjD symptoms (72%) indicated that the symptoms were present for six to twenty-four months (Maercker et al., 2012). Finally, a study assessing AjD symptoms several years after organ transplantations found that the time since the medical procedure was unrelated to AjD symptom severity (Bachem, Baumann, & Köllner, 2019). To the best of our knowledge, these are the only studies that specifically focused on the course of AjD over time based on a recent definition of the disorder, all of them putting the six months’ period in question.

The Zurich Adjustment Disorder Study (ZADS) investigates the validity of the new ICD-11 definition of AjD in a sample of individuals who involuntarily lost their job and explores predictors of AjD development over time. Previous analyses revealed that the prevalence of AjD in this high-risk sample was 15.5% when applying the full ICD-11 diagnostic criteria to a structured diagnostic interview schedule (Perkonigg, Lorenz, & Maercker, 2018). Based on questionnaire results, the prevalence of a tentative AjD diagnosis was 25.6% at approximately three months after the last day at work (Lorenz, Perkonigg, & Maercker, 2018b), and 18.2% six months later (Lorenz, Makowski, & Maercker, 2019).

Demographic factors such as higher age, female gender or low household budget as well as characteristics of the stress experience such as first job loss, a job that required “brainwork”, a job with high responsibility, and a larger number of job applications written to get a new position correlated with higher symptom severity and/or higher odds for a diagnosis of AjD (Perkonigg et al., 2018). Established intrapersonal resources that support coping with adversity such as high self-efficacy and sense of coherence were similarly related to fewer symptoms of AjD (Perkonigg et al., 2018). Finally, the socio-interpersonal framework model for stress-response syndromes (Maercker & Horn, 2013) suggests that different levels of social contexts play a crucial role in the recovery after stress experiences. These contexts include social affects (e.g., shame, anger, loneliness), interactions in close relationships (e.g., social support, empathy) or societal and cultural factors (e.g. social acknowledgement). In accordance with the model, lower self-efficacy, stronger feelings of loneliness, higher dysfunctional disclosure, less perceived social support, and more negative social interactions were identified as correlates of higher symptom severity (Lorenz, Perkonigg, & Maercker, 2018b).

The aim of the present paper is to expand upon previous findings of the ZADS and other longitudinal investigations. First, we aimed to report on the development of AjD symptoms and ICD-11 core symptom clusters in the context of involuntary job loss across three assessments. Based on the current disorder model and previous research, we expected that the prevalence rates of symptoms and symptom clusters would be high initially and that they would decline after six and twelve months. Second, several potential predictors of AjD development were explored. We hypothesized that AjD-related features (initial AjD symptoms, life events experienced), socio-demographic factors (gender, age, household income), and psychosocial factors relevant for stress-response syndromes (e.g., personal beliefs, interpersonal resources) would be associated with long-term outcome.

## Method

### Participants and Procedure

**Figure 1:**
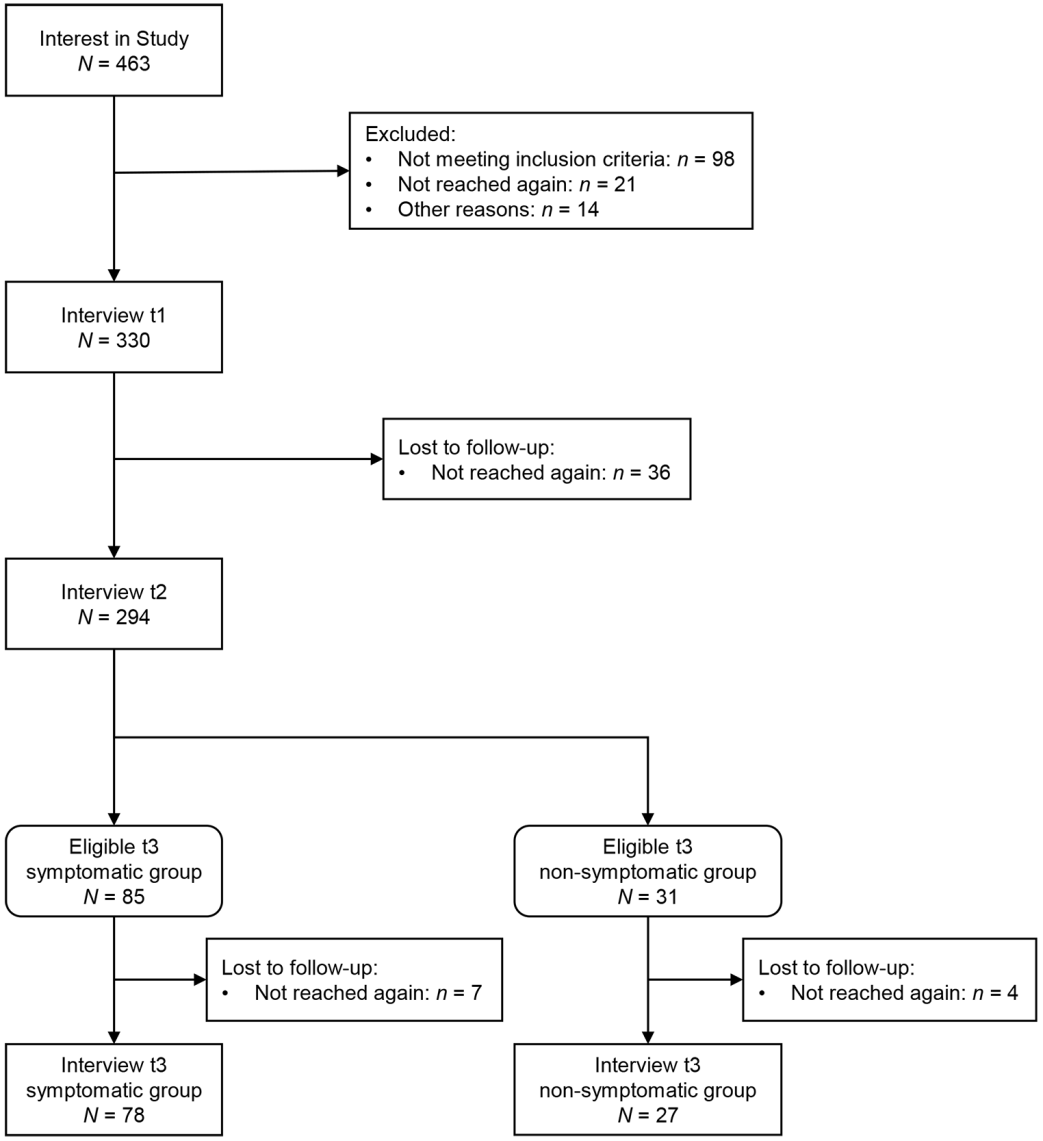
Participant Flow of the Zurich Adjustment Disorder Study *Note.* t1 = first assessment; t2 = second assessment; t3 = third assessment.

The data for the present analysis stem from the ZADS investigating the new proposal for adjustment disorder in ICD-11 in a sample of individuals who experienced involuntary job loss (Perkonigg et al., 2018). The Ethics Committee of the University of Zurich approved the study in June 2015 and all participants gave written informed consent. The study included all participants who were assessed at three time points with a fully structured clinical diagnostic interview for AjD. The first assessment took place up to nine months after the last day at work (t1), followed by a six-months (t2) and a twelve-months (t3) follow-up assessment. The participants were recruited through local employment offices, newspaper articles, and mailing lists in the greater Zurich area. Participants were excluded if they did not speak German fluently, were unable to give written informed consent, or suffered from a severe mental illness. The latter criterion led to the exclusion of one individual who was assumed to experience a psychotic episode. All participants were invited to participate in the first and second assessment of the study. Since a comparison of extreme groups was planned for the original study, only a sub-sample was invited to the third assessment. Inclusion in the sub-sample was determined after completion of t2. In the *symptomatic group*, we invited individuals who (a) met the criteria for an AjD at t1 or a subclinical AjD (either only preoccupation or only failure to adapt) at t1 and who (b) identified the same worst event at t1 and t2. In the *non-symptomatic group*, we invited individuals who reported a maximum of one symptom of AjD at t1 and at t2. Of the 330 individuals that participated in the first assessment, 294 took part in the second assessment. Of these individuals, 78 met the criteria for the symptomatic group and could be assessed a third time; 27 individuals met the criteria for the non-symptomatic group and could be assessed a third time. This led to a total sample size of *N =* 105 for the present analysis. The participant flow is shown in Figure 1.

Table 1 displays a summary of the demographic characteristics of the sample. T1 was conducted *M* = 3.4 (*SD* = 2.1) months after the last day at work (*Mdn* = 3.2). The interval between t1 and t3 was *M* = 12.3 (*SD* = 0.8) months. At t3, 17.1% (*n* = 18) of the participants had started a new job since t2, 48.6% (*n* = 51) of the sample continued the new job they had started between t1 and t2, 30.5% (*n* = 32) were still unemployed, and 1.9% (*n* = 2) experienced a new job loss.

**Table 1:** Demographic Characteristics of the Sample (N = 105)

Variable	*M*	*SD*	*N*	%
**Age at t1**	46.3	10.0		
Gender				
Male			56	53.3
Female			49	46.7
Marital status at t1				
Married			38	36.2
Separated / divorced			21	20.0
Never married			45	42.9
Registered partnership			1	1.0
**Children at t1**	0.9	1.1		
Vocational qualification				
On-the-job-training			3	2.9
Formal apprenticeship			39	37.1
University / University of applied sciences			56	53.3
PhD			3	2.9
No qualification			2	1.9
Missing			2	1.9

### Measures

#### Adjustment Disorder Module for Composite International Diagnostic Interview (AjD-CIDI)

Adjustment disorder was assessed with a new module of the Composite International Diagnostic Interview (CIDI) that specifically focuses on AjD after ICD-11 and DSM-5 (AjD-CIDI) (Perkonigg, Strehle, et al., in press). In the beginning, the AjD-CIDI assesses stressors (e.g. family conflict, financial problems, illness of a loved one) that occurred during the 12 months prior to the interview and event-specific characteristics (e.g. time of onset, duration). At the end of this first part, the participants were asked which of the events they experienced as the most distressing. The second part of the interview asks for a range of symptoms occurring in response to this event following the ICD-11 and the DSM-5 definition. The 25 symptoms represent the areas of preoccupation with the stressor and failure to adapt to the stressor, as well as accessory symptoms of avoidance, depression, anxiety and impulsivity. The third part of the module assesses information about onset, recency of symptoms and functional impairment (Perkonigg, Strehle, et al., in press).

We used a modified follow-up version of the AjD module for t2. In this version, the first part asks for new life events and the most distressing event from the previous interview is coded. The participant then indicated the currently most distressing event out of the new and the old events. Then, the second and third part of the AjD-CIDI were applied with regard to the event coded at t1. At t3, the symptomatic group was interviewed with a version that asked specifically for symptoms in response to the event they talked about at t1 and t2.

The diagnosis of AjD according to ICD-11 (WHO, 2018) was made if the following criteria were met: A) occurrence of a significant life event; B) presence of at least one symptom of preoccupation (recurrent involuntary thoughts about the event, and constant worries related to the event); C) presence of at least two failure to adapt symptoms (concentration problems, difficulties at work/daily activities, loss of interest in work, social network or leisure activities, sleep problems, and loss of self-confidence); D) frequency of symptoms at least 10-15 times per month or clinical relevance of symptoms (impairment at least “moderate” or contact with a health professional about the symptoms); E) exclusion of cases who presented with a current depressive episode and of cases who presented with a current generalized anxiety disorder as defined by the CIDI.

#### Scales for Predictor Variables

The *General Self-Efficacy Scale* (GSE; Schwarzer & Jerusalem, 1999) was used for the assessment of self-efficacy. The 10-item scale has a 4-point Likert scale response-format (1, *not correct* – 4, *absolutely correct*). The total score is obtained by summing up all individual items and higher scores indicate higher self-efficacy. The psychometric properties of the GSE were satisfactory in earlier validation studies with internal consistencies of .80 – .90 (Hinz, Schumacher, Albani, Schmid, & Brähler, 2006; Schwarzer & Jerusalem, 1999). The internal consistency in the present study was α = .88.

We measured sense of coherence using the *Sense of Coherence Scale – Revised* (SOC-R; Bachem & Maercker, 2018). The scale, consisting of 13 items, measures manageability, reflection, and balance. The response-format is a 5-point Likert scale (1, *not at all*, - 5, *completely)*. All items are summed up to build a total score of the SOC-R, with one recoded item. Higher scores indicate a higher sense of coherence. Earlier validation studies reported satisfactory psychometric properties for the SOC-R with internal consistencies of α = .75 – .81 (Bachem & Maercker, 2018; Mc Gee, Höltge, Maercker, & Thoma, 2018). The internal consistency in the present study was α = .71.

A composite score of two single items from other scales was used to measure *feelings of loneliness* (Lorenz, Perkonigg, & Maercker, 2018b). We used one item from the Brief Symptom Inventory – 18 (Spitzer et al., 2011) and one item of the Social Functioning Questionnaire (Tyrer et al., 2005). The item formulations were *‘How strong did you experience feelings of loneliness during the past 7 days?’* and *‘I feel lonely and isolated from other people’*. The response-format was a 5-point Likert scale (0, *not at all* – 4, *very strong*) and a 4-point Likert scale (0, *almost all the time* – 3, *not at all*), respectively. The latter item was recoded before building a sum score with the first item of the scale. The correlation between the two items in the present study was α = .70.

The *Disclosure of Trauma Questionnaire* (DTQ) was used in an abbreviated form (Pielmaier & Maercker, 2011) to measure dysfunctional disclosure. The scale, consisting of 12 items with a 6-point Likert scale (0, *not at all* – 5, *absolutely*) response-format, measures the urge to talk, the reluctance to talk, and emotional reactions while disclosing. The individual items are summed up to build a total score; higher scores indicate higher dysfunctional disclosure. Previous studies found satisfying psychometric properties for the DTQ (Müller, Beauducel, Raschka, & Maercker, 2000; Müller & Maercker, 2006). The internal consistency of the abbreviated form was α = .75 in previous studies (Pielmaier & Maercker, 2011) and α = .81 in the present study.

We used the *Social Support Questionnaire, short form* (FSozU-K; Fydrich, Sommer, Tydecks, & Brähler, 2009) to measure perceived social support. The 14 items are answered on a 5-point Likert scale (1, *don’t agree*, - 5, *agree)*. The mean of all answered items is used to build the total score and higher scores indicate higher perceived social support. The psychometric properties in the validation of the FSozU-K were satisfactory with an internal consistency of α = .94 (Fydrich et al., 2009). The internal consistency in the present study was α = .93.

A subset of items of the Daily Hassles Scale (Perkonigg & Wittchen, 1998) was used to measure *negative social interactions* (Lorenz, Perkonigg, & Maercker, 2018b). Six items measured negative interactions with the partner, children, parents, siblings, friends, or neighbours during the last two weeks. The original 4-point Likert scale response-format of the items (1, *often* – 4, *never*) was reverse coded, so that a higher mean score indicates more negative social interactions. The internal consistency was α = .68 in a previous study (Lorenz, Perkonigg, & Maercker, 2018b) and α = .73 in the present study.

The *Social Acknowledgement Questionnaire* (SAQ; Maercker & Müller, 2004) measured perceived acknowledgement of the difficult situation of the individual by the social environment. The 16 items, answered on a 4-point Likert scale (0, *not at all* – 3, *completely*), measure general disapproval, disapproval by family or friends, and recognition as a victim. Following the authors of the scale, the total score was built by summing up items 3, 9, and 11 through 16, and subtracting items 1, 2, 4 through 8, and 10. A higher score indicates more social acknowledgement. The validation study of the questionnaire reported satisfactory psychometric properties with an internal consistency of α = .86 (Maercker & Müller, 2004). The internal consistency in the present study was α = .73.

### Statistical Analysis

Data were analysed using SPSS version 23. The highest number of missing values was found for social acknowledgement (13%), all other variables had less than 3% missing values and data were missing completely at random. Pairwise case deletion was used in the analyses. The prevalence of ICD-11 AjD was computed with and without consideration of the exclusion criterion. To investigate predictive factors, we performed a hierarchical regression analysis with the number of symptoms at t3 as outcome. We decided to include all symptoms that were measured by the AjD-CIDI to increase the variance of the outcome variable and because there is still uncertainty about the best conceptualisation of AjD (Lorenz, Hyland, Perkonigg, & Maercker, 2018). The analysis included three steps. In the first step, we included the number of symptoms at t1, the total number of life events reported at t1, and the total number of new life events reported between t1 and t3 as predictors. The second step included socio-demographic characteristics (gender, age, household income < 4000 Swiss francs) and the third step included psychosocial variables (general self-efficacy, loneliness, dysfunctional disclosure, perceived social support, negative social interactions, social acknowledgement). In the second and third step, we included predictor variables that were found to be associated with initial symptom severity and 6-months outcomes in previous publications from this sample (Lorenz, Hyland, et al., 2018; Lorenz, Perkonigg, & Maercker, 2018a, 2018b; Perkonigg et al., 2018). The final model was selected based on the significance of the *F*-statistics. No multicollinearity was found based on the VIF measure (ranged between 1.030 and 1.078).

## Results

### Descriptives

The total amount of symptoms as measured by the AjD-CIDI was *M* = 7.1 (*SD* = 5.5; *Mdn* = 7.0, *range* = 0-19) at t1, *M* = 4.3 (*SD* = 5.0; *Mdn* = 2.0, *range* = 0-20) at t2, and *M* = 2.1 (*SD* = 2.8; *Mdn* = 1.0, *range* = 0-13) at t3. The total number of life events reported at t1 was *M* = 2.3 (*SD* = 1.2, *range* = 1-7) and the total number of new life events experienced between t1 and t3 was *M* = 1.0 (*SD* = 1.3, *range* = 0-7). The majority of participants (74.3%) indicated the job loss, financial problems or problems with authorities as their worst event at t1, followed by family matters (22.9%; family conflicts/separation/illness or death of family member). The descriptive statistics for the predictor variables and the correlation coefficients between the main predictor variables can be found in the supplementary material.

### Prevalence of AjD Symptoms

**Figure 2:**
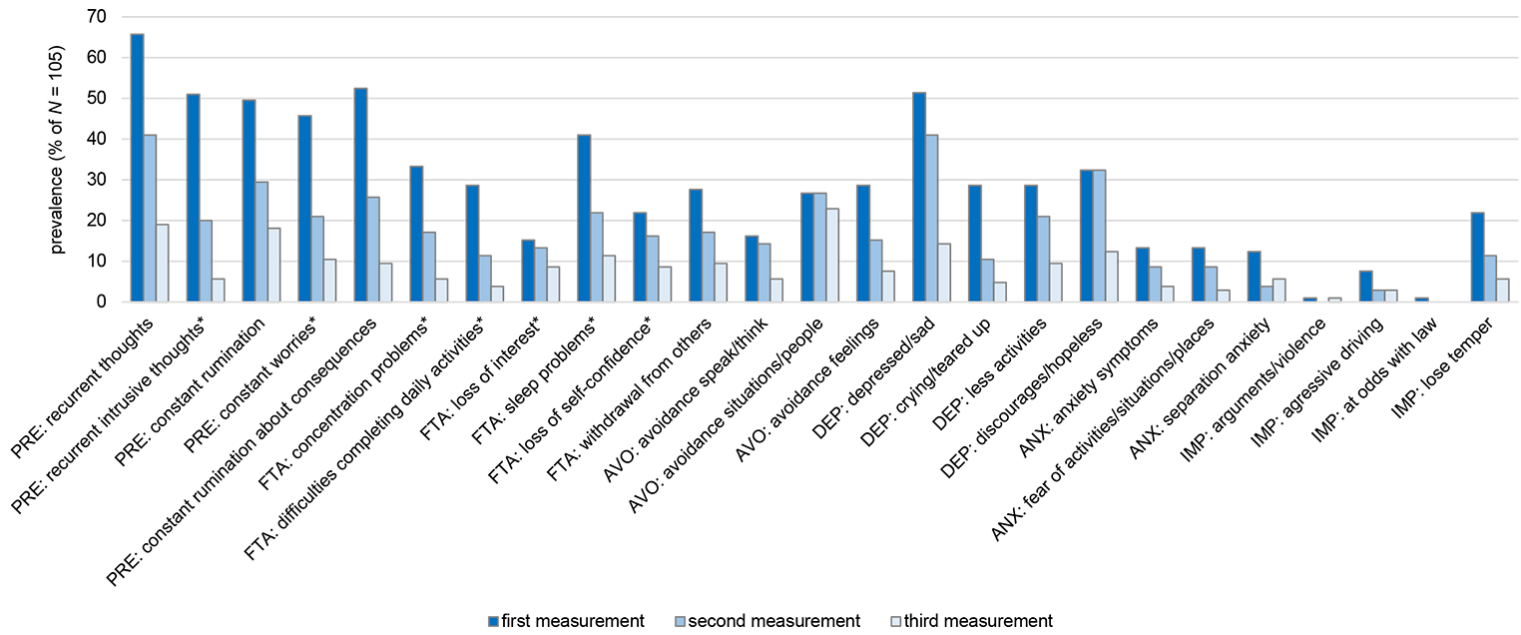
Prevalence (%) of Individual Symptoms That May Occur in ICD-11 Adjustment Disorder Across the Three Assessments *Note.* PRE = Preoccupation; FTA = Failure to adapt; AVO = Avoidance; DEP = Depression; ANX = Anxiety; IMP = Impulsivity. *items used for diagnostic algorithm for adjustment disorder.

The prevalence rates of the individual symptoms as measured by the AjD-CIDI are displayed in Figure 2. For the majority of symptoms, the prevalence was highest at t1 and lowest at t3. The symptoms measuring preoccupation with the stressor, sleep disturbances (as part of failure to adapt), and feeling low and sad (as part of depressive symptoms) were the most prevalent at t1 with over 40% of the individuals reporting each of them. At t2, repetitive thoughts, feeling low and sad, and feeling discouraged and hopeless for the future (depressive symptom) were the most prevalent symptoms (each over 30%). The most prevalent symptoms at t3 were repetitive thoughts, rumination about the event, and avoiding situations or individuals that could remind of the event (avoidance symptom) with roughly a 20% prevalence each.

### Prevalence of AjD Symptom Groups

Table 2 displays the prevalence of the diagnostic criteria across the three assessments. Criterion A was met by every participant since the presence of a stressor was an inclusion criterion of the study. The prevalence rates of preoccupation (Criterion B), failure to adapt (Criterion C), and impairment in social functioning (Criterion D) were highest for the first assessment and declined over time. The prevalence rate of exclusive disorders (Criterion E) remained stable across the three assessments. Approximately every fifth individual met the full diagnostic criteria at t1 (21.9%). This prevalence declined to 6.7% at t2, and to 2.9% at t3. The majority of individuals reported no AjD across all assessments (*n* = 80; 76.2%). Most of the other participants met the diagnostic guidelines only at t1 (*n* = 16, 15.2%) or only at t1 and t2 (*n* = 5, 4.8%). One individual (1.0%) received an AjD diagnosis at all three assessments.

**Table 2:** Prevalence of Adjustment Disorder Criteria Across the Three Assessments

Adjustment Disorder Criterion	T1		T2		T3	
	*N*	%	*N*	%	*N*	%
Criterion A: Event	105	100.0	105	100.0	105	100.0
Criterion B: Preoccupation	63	60.0	32	30.5	15	14.3
Criterion C: Failure to adapt	44	41.9	18	17.1	10	9.5
Criterion D: Impairment	82	78.1	67	63.8	40	38.1
Criterion E: Exclusive disorders	10	9.5	10	9.5	9	8.6
ICD-11 Adjustment disorder without exclusion criterion	29	27.6	12	11.4	4	3.8
ICD-11 Adjustment disorder with exclusion criterion	23	21.9	7	6.7	3	2.9

*Note.* T1 = first assessment; T2 = second assessment; T3 = third assessment.

### Prediction of AjD Symptoms at t3

Table 3 displays the results of the hierarchical regression analysis for the total number of AjD-CIDI symptoms at t3. The first step included the number of AjD-CIDI symptoms at t1, the number of life events reported at t1, and the number of new live events experienced between t1 and t3 as predictors. This model was significant, *F*(3, 86) = 7.648, *p* < .001. The second model, which included socio-demographic characteristics, and the third model, which included psycho-social variables, did not significantly increase the fit of the model. Thus, the model only including adjustment disorder related characteristics (Model 1) was interpreted. A higher number of AjD-CIDI symptoms at t1 and a higher number of life events experienced between t1 and t3 were associated with a higher number of AjD-CIDI symptoms at t3. The model explained 18% of the variance in the outcome (adjusted *R*^2^ = .183).

**Table 3:** Hierarchical Regression Results (Standardized β Coefficients) for the Total Number of AjD-CIDI Symptoms at the Third Assessment (N = 105)

Predictor	Model		
	1	2	3
Number of AjD-CIDI symptoms at t1	0.316**	0.365***	0.278*
Number of life events at t1	0.060	0.083	0.088
Number of new life events between t1 and t3	0.291**	0.286**	0.292**
Gender		-0.235*	-0.205
Age (t1)		0.046	0.007
Household income < 4000 SFr (t1)		0.000	-0.001
General self-efficacy (t1)			-0.079
Sense of coherence (t1)			-0.029
Loneliness (t1)			0.164
Dysfunctional disclosure (t1)			-0.032
Perceived social support (t1)			0.078
Negative social interactions (t1)			0.069
Social acknowledgement (t2)			-0.035
*F*	7.648***	2.130	0.518
*R* ^2^	.211	.267	.300
adjusted *R*^2^	.183	.214	.181
Δ*R*^2^		.056	.033

*Note.* Gender: 1 = male; 2 = female; Household income < 4000 SFr (0 = no; 1 = yes).

*p < .05. **p < .01. ***p < .001.

## Discussion

The aim of the present analysis was to investigate the course of adjustment disorder in the context of involuntary job loss over the course of twelve months. It was the first investigation of prevalence rates according to ICD-11 with a new structured diagnostic interview in a high-risk sample. We found an AjD prevalence rate of 21.9% at the first assessment. Previous studies using ICD-10 or DSM-IV criteria found prevalence rates ranging between 6.9% and 38% in high risk populations (e.g., Mitchell et al., 2017; Rundell, 2006), between 3% and 12% in medical settings (e.g., Fernández et al., 2012; Yaseen, 2017), and between 11% and 17% in psychiatric settings (Bruffaerts, Sabbe, & Demyttenaere, 2004; Shear et al., 2000). Based on a self-report questionnaire, studies investigating the new ICD-11 approach reported varying prevalence rates between 21% and 61% in high-risk populations (e.g., Dannemann et al., 2010; Dobricki, Komproe, de Jong, & Maercker, 2010). However, they refer to a tentative diagnosis and did not apply the ICD-11 exclusion criterion. The prevalence rate in this sample, consisting of extreme groups with high or low AjD symptoms at previous assessments, dropped to 3% at the third assessment, which is only slightly higher than prevalence rates found in general population-based samples (e.g., Ayuso-Mateos et al., 2001; Glaesmer, Romppel, Braehler, Hinz, & Maercker, 2015). At the same time, the prevalence rate was lower than the twelve-months prevalence rate found in the O’Donnell et al. (2016) study investigating the DSM-5 model in a post-injury sample. This could be either an effect of the different diagnostic guidelines applied (ICD-11 or DSM-5) or an effect of the stressor (job loss vs. injuries). Future studies should aim at a direct comparison between ICD-11 and DSM-5 diagnostic guidelines.

As expected, there was a decline in AjD symptoms over time. This generally supports the assumption of a favourable outcome of AjD. However, a substantial proportion (seven of the twenty-three cases) with an AjD at the first assessment still met the diagnostic criteria for an AjD six months later. This represents 30% of the AjD cases that show a longer duration of the disorder than the conditional six-month threshold in ICD-11 and DSM-5. It could be argued that the life event ‘job loss’, which was rated to be the worst event by the majority of the sample, or its consequences is often not resolved within the time period of six months the ICD-11 mentions as “typical” for a resolution. This argument is supported by the high number of new or subsequent life events in the present sample, which might complicate recovery. It emphasizes the difficulty of applying time period features like six months in stress-related disorders and implies to use this feature only after a thorough substantive examination and a flexible interpretation of the abovementioned period.

The second aim of this study was to investigate factors that predict AjD symptoms after twelve months. The hierarchical approach allowed us to examine whether only AjD-related characteristics explain long-term outcome or whether socio-demographic factors and psychosocial processes add explanatory power over the course of twelve months. The results indicate that higher initial symptomatology and more life stressors following the event significantly predicted higher symptomatology twelve months later and that AjD-related characteristics might be a sufficient explanation for symptom severity over the course of twelve months, supporting the concept of a stress-response syndrome. However, the selection of potential risk and protective factors was limited, and future studies should include other relevant predictors since the model was only able to explain 18% of the variation in symptom severity after twelve months.

We included socio-demographic and psychosocial predictors that were associated with initial symptom severity in earlier studies (e.g., Lorenz, Perkonigg, & Maercker, 2018b; Perkonigg et al., 2018). Although these predictors were not longitudinally associated with AjD symptoms, they were associated with initial symptom severity. Since initial symptom severity was one of the strongest predictors of long-term outcome, the effect of the socio-demographic and psychosocial predictors on t3 AjD symptoms could be indirect, via symptoms at t1. Hence, future studies could focus on a possible mediation effect of initial symptom severity on the association between socio-demographic and psychosocial predictors and long-term outcome. If this mediation was true, it could be reasonable to target these factors to achieve a better long-term outcome. This assumption finds support in two recent self-help intervention studies for AjD. These interventions aimed at enhancing resilience for example by improving problem-solving skills or mobilizing social support and showed medium to large effect sizes for the reduction of AjD related symptomatology over time (Bachem & Maercker, 2016; Eimontas, Rimsaite, Gegieckaite, Zelviene, & Kazlauskas, 2018). Alternative explanations for the result that especially the number of life events predicted symptom severity at t3 could be memory effects or attention deficits. The AjD-CIDI stressor list also covers psychosocial stress of minor intensity, such as troubles with neighbours or giving up a hobby. Individuals who are worse off could be particularly sensitive to these minor stressors while better adjusted individuals may find it unnecessary to report these events.

The analyses for AjD symptoms were based on all symptoms that may occur in AjD rather than only the ICD-11 core symptom cluster of preoccupation and failure to adapt because of the differences between the major diagnostic classification systems. While the ICD-11 defines specific core symptoms (WHO, 2018), the DSM-5 kept the previous definition that is not based on specific criteria but on the exclusion of other mental disorders (APA, 2013). These dissimilarities are a result of the lack of research around AjD and of a lack of agreement on the main characteristics of the disorder, and they might result in differences in access to treatment. Across the three assessments, different symptoms of preoccupation with the stressor were among the most prevalent symptoms, supporting the inclusion of this symptom group in the diagnostic guidelines in ICD-11. Symptoms that reflect depressive reactions were also commonly present, suggesting that it might be reasonable to include mood alterations in the AjD definition as it is the case in DSM-5. These results could be a first evidence for the validity of both approaches and further revisions of the guidelines might include features of both definitions. Future research should not only focus on the most prevalent symptoms but also try to identify symptoms that are associated with high functional impairment or that show high discriminatory power.

The use of the new ICD-11 diagnostic guidelines and a fully structured clinical diagnostic interview make this study unique. Still, it has several limitations. First, the data stems from a particular high-risk sample, which limits the generalizability to all AjD cases. Second, the sample for this study was based on specific selection criteria. We specifically defined a symptomatic and a non-symptomatic group to increase variance in the data. Moreover, we lifted inclusion criterion b) for the non-symptomatic group in order to be able to investigate incidence rates for adjustment disorder. This specific methodology complicated interpretation of prevalence findings at t3. Furthermore, the recruitment was based on self-selection since we did not apply a systematic or stratified recruitment strategy. These methodological concerns restrict the generalizability of the results to the whole population of unemployed individuals. Third, we did only control for the presence of a depressive episode and/or generalised anxiety disorder and not the full list of exclusive disorders as recommended by ICD-11. Future studies should consider the full range of clinically meaningful exclusions. Fourth, the interval between assessments was chosen at six months to investigate the proposal of the diagnostic guidelines for AjD. Research that includes shorter intervals between assessments could shed further light into the dynamics of the disorder. Last, the number of predictors in the hierarchical regression could have limited the power of the analysis considering the sample size. This could have masked some predictive effects and future studies should increase the sample size. In addition, loneliness was assessed with two items from different scales rather than with an established questionnaire.

Adjustment disorder has been a diagnostic category that received little attention in research despite a frequent use in clinical practice (Evans et al., 2013; Reed, Correia, Esparza, Saxena, & Maj, 2011). The relatively high prevalence of AjD in this study, the methodological concerns raised by our findings, and the aforementioned issues of disorder definition again stress the importance of a systematic inclusion of AjD in research in order to understand maladaptive responses to life stress better, especially since AjD is associated with a higher risk for the development of severe psychopathology and suicidality (e.g., Casey & Doherty, 2012; O’Donnell et al., 2016). This study furthermore showed that even though AjD symptomatology shows a favourable course over time, it can also persist beyond the six-month threshold as proposed by ICD-11 and DSM-5. Further research is needed to understand the mechanisms underlying the disorder and determining the long-term outcome of AjD. Moreover, future studies comparing prevalence rates between ICD-11 and DSM-5 may deepen our understanding of maladjustment to stressful life events.

## Supplementary Materials

The Supplementary Materials contain the descriptive statistics of the main measures of the study and the correlations between study variables (for access see Index of Supplementary Materials below).

10.23668/psycharchives.3463 Supplement 1 Supplementary materials to "The 12-month course of ICD-11 adjustment disorder in the context of involuntary job loss"

LorenzL.
MaerckerA.
BachemR.
 (2020). Supplementary materials to "The 12-month course of ICD-11 adjustment disorder in the context of involuntary job loss"
[Descriptive statistics and correlation coefficients]. PsychOpen. 10.23668/psycharchives.3463PMC964547936398147

## Data Availability

Data from this study are not publicly available as informed consent and ethical approval for public data sharing were not obtained from participants. The data are readily available upon request by qualified scientists. Any enquiries regarding data accessibility can be addressed to the first author.
