# Supplementary material for: The 12-Month Course of ICD-11 Adjustment Disorder in the Context of Involuntary Job Loss
Source: Clin Psychol Eur. 2020 Sep 30;2(3):e3027. doi: 10.32872/cpe.v2i3.3027 (PMC9645479; doi:10.32872/cpe.v2i3.3027)
Supplement: Supplement 1 [file cpe-02-3027-s1.pdf]

The 12-Month Course of ICD-11 Adjustment Disorder Following Involuntary Job Loss

Supplementary Tables

Louisa Lorenz[a]*, Andreas Maercker[a], and Rahel Bachem[a]

[a]Department of Psychology, University of Zurich

Binzmuehlestrasse 14/17, CH-8050 Zurich, Switzerland

***Corresponding author:**

l.lorenz@psychologie.uzh.ch, 0041 44 635 74 57

Supplementary Table 1

Descriptive statistics of the main measures for the whole sample and divided by gender

	Full sample (N = 105)		Male (n = 56)		Female (n = 49)		Gender Effect
	M	*SD*	*M*	*SD*	*M*	*SD*	*d*
Number of AjD-CIDI symptoms at t3	2.10	2.85	2.50	3.17	1.63	2.37	0.31
Number of AjD-CIDI symptoms at t1	7.05	5.47	5.96	4.70	8.29	6.05	-0.44
Number of life events at t1	2.30	1.24	2.16	1.14	2.45	1.34	-0.23
Number of new life events between t1 and t3	1.00	1.29	1.00	1.25	1.00	1.34	0.00
Age (t1)	46.33	10.04	47.29	9.43	45.24	10.69	0.20
General self-efficacy (t1)	31.33	4.33	31.29	4.63	31.37	4.02	-0.02
Sense of coherence (t1)	51.96	5.32	52.39	5.83	51.49	4.70	0.17
Loneliness (t1)	1.27	1.42	1.20	1.37	1.35	1.48	-0.11
Dysfunctional disclosure (t1)	14.61	9.03	13.29	7.44	16.15	10.46	-0.32
Perceived social support (t1)	4.33	0.70	4.31	0.64	4.36	0.77	-0.04
Negative social interactions (t1)	1.72	0.50	1.63	0.47	1.84	0.50	-0.43
Social acknowledgement (t2)	3.25	6.16	3.23	6.07	3.27	6.32	0.00

Note: T1 = first measurement; T2 = second measurement; T3 = third measurement.

Supplementary Table 2

Correlation between Study Variables (Pearson coefficient) (N = 105)

	1.	2.	3.	4.	5.	6.	7.	8.	9.	10.	11.	12.
1. Number of AjD-CIDI Symptoms at t3	-	.34***	.17	.32**	.03	-.25**	-.07	.31**	.22*	-.12	.17	-.19
2. Number of AjD-CIDI symptoms at t1		-	.18	.05	-.06	-.38***	-.22*	.48***	.55***	-.27**	.43***	-.29**
3. Number of life events at t1			-	.17	-.08	.09	.23*	.16	.23*	.15	.17	-.01
4. Number of new life events between t1 and t3				-	-.03	-.08	.07	.03	.15	.02	.15	-.07
5. Age (t1)					-	-.13	.11	.09	-.02	.09	-.05	.00
6. General self-efficacy (t1)						-	.31**	-.36**	-.15	.31**	-.47**	.25*
7. Sense of coherence (t1)							-	-.20*	-.12	.32**	-.05	.05
8. Loneliness (t1)								-	.40**	-.44**	.32**	-.37**
9. Dysfunctional disclosure (t1)									-	-.19	.32**	-.33**
10. Perceived social support (t1)										-	-.26**	.37**
11. Negative social interactions (t1)											-	-.32**
12. Social acknowledgement (t2)												-

Note: T1 = first measurement; T2 = second measurement; T3 = third measurement.
